# Puerperal Fever—Its Causes and Modes of Propagation

**Published:** 1857-11

**Authors:** 


*Puerperal Fever—its Causes and Modes of Propagation. Read before the New York Academy of Medicine, at the opening of the discussion on Puerperal Fever, on the 4th of April, 1857, and published by consent of that body. By Joseph M. Smith, M.D., Professor of Mat. Medica and Clin. Med. in the New York College of Physicians and Surgeons, Physician to the N. York Hospital, etc. etc. From the New York Journal of Medicine for Sept. 1st, 1857.*

Dr. Smith rejects the idea of contagiousness of puerperal fever, and supplies the explanation of the many circumstances that favor such an opinion, by supposing that it is produced by idio-miasma, "generated by the foul discharges of puerperal women in crowded, ill-ventilated lying-in hospitals; sometimes from the absorption of putrescent matters lodged in the uterus and vagina after parturition; sometimes from the exhalations of patients laboring under typhus fever, erysipelas and gangrenous diseases; and sometimes from the emanations from the human body dissected after death. It further appears," he says, "that the miasms of typhus, erysipelas and puerperal fever are severally capable of producing any one or all of these diseases, and that they may attach themselves to the persons or clothing of midwives and physicians, and thus be transported from their sources to the chambers of the lying-in women. It is also observable that the more ordinary form of disease, induced by the febrific effluvia in question, is typhus and its modification, typhoid fever, whilst puerperal and hospital erysipelas are but varieties of that disease, taking their forms from the peculiar predisposing conditions of the system and certain epidemic influences." To prevent it, ventilation, fumigation, cleanliness of the surrounding and dispersion of the patients, and the entire cessation from practice of the physician in whose care such cases prevail. While we are willing to acknowledge that Dr. Smith has entirely established every point in the above ingenious, readable, learned and instructive pamphlet, we cannot help but admire, with almost astonishment, the accomodating elasticity of the two hypothetical entities, contagion and miasm. We have often witnessed their permeating expansibility—first one and then the other—under the warm enthusiasm of genius, pervading and occupying every labyrinth and intricacy of etiology, and supporting with powerful buoyancy the most ponderous theories, and alternately (but not conjointly) occupying the same space. We have been led by one observer through a world of facts, proving incontestibly that there is *no* contagion, while another has pointed to incontrovertible evidences all around of the fact salient in obviousness, that there are almost no diseases but what, under certain circumstances, may become contagious, until we almost despair of ever seeing this "vexed question" settled.

Dr. Smith's essay is an excellent one of its kind, and will well repay any who may take time to read it carefully. It is replete with learning and ingenuity, and is unexceptionable in style, while the subject is absorbingly interesting.

